# quaqc: efficient and quick ATAC-seq quality control and filtering

**DOI:** 10.1093/bioinformatics/btae649

**Published:** 2024-10-30

**Authors:** Benjamin J M Tremblay, Julia I Qüesta

**Affiliations:** Centre for Research in Agricultural Genomics (CRAG), CSIC-IRTA-UAB, Bellaterra, Barcelona, 08193, Spain; Centre for Research in Agricultural Genomics (CRAG), CSIC-IRTA-UAB, Bellaterra, Barcelona, 08193, Spain

## Abstract

**Summary:**

“quaqc” allows for ATAC-seq-specific quality control and read filtering of NGS data with minimal processing time and extremely low memory overhead. An efficient scaling implementation allows for a wide range of use cases, from processing individual samples processed on personal laptops to handling thousands of samples processed in parallel on compute clusters. The helper R package “quaqcr” allows for interactive program execution and exploration of results.

**Availability and implementation:**

Source code and documentation are freely available for download from https://github.com/bjmt/quaqc and https://github.com/bjmt/quaqcr under the GPLv3 license. “quaqc” is implemented in C and has been tested on both macOS and Linux. The “quaqcr” helper package only requires the R programming language. Fixed versions of the programs and code associated with this manuscript can be found at https://zenodo.org/records/13833437.

## 1 Introduction

Profiling accessible chromatin regions (ACRs) is an important aspect of understanding eukaryotic gene regulation, providing insights into regions of the genome, which are involved in transcription factor regulation of gene expression (such as promoters and enhancers) (Klemm *et al.* 2019). Various methods have been used to this effect, including DNase-seq (Song and Crawford 2010), FAIRE-seq (Giresi *et al.* 2007), ATAC-seq (Buenrostro *et al.* 2013), and others. Of these methods ATAC-seq is by far the most widely used, due to its high signal-to-noise ratio, low cost, small sample requirement, and short protocol time (Grandi *et al.* 2022). In this method, isolated nuclei or cell suspensions are incubated with a Tn5 transposase which inserts short oligos into DNA, which will preferentially act upon exposed DNA accessible in regions of open chromatin (Buenrostro *et al.* 2013). Fragments of DNA, containing the inserted oligo on both 5ʹ and 3ʹ ends, can be amplified using matching primers with sequencing adapter overhangs thus generating a DNA sequencing library in a single step. A final size selection is generally required for the removal of unused adapter primer and overly short fragments before sequencing.

Successful preparation of high-quality ATAC-seq libraries can be impeded by a number of circumstances, including improper library size selection, low quality nuclei, the presence of free DNA in the reaction mix, a high proportion of non-nuclear DNA (e.g. mitochondria, chloroplasts), and incorrect nuclei quantity to Tn5 transposase ratio, among others (Orchard *et al.* 2020, Zhang *et al.* 2022). These factors can lead to insufficient read counts in target nuclear sequences and high levels of background noise. Several quality control checks can be employed to detect such events in the final sequencing data, and proper read filtering can minimize their impact (Yan *et al.* 2020). Several tools are currently used for the purpose of quality control, including “ataqv” (Orchard *et al.* 2020) and the R package “ATACseqQC” (Ou *et al.* 2018), though general solutions not specific to ATAC-seq data such as “Picard Tools” (Broad Institute 2024) are also used. Read filtering is typically performed as a separate step with tools such as “Picard Tools” and “samtools” (Li *et al.* 2009, Danecek *et al.* 2021).

Here, we introduce “quaqc,” a program for simultaneous comprehensive quality control and read filtering of ATAC-seq data. This all-in-one tool combines many important features that are either missing or not found together in other existing tools, including simultaneous proper handling of mitochondrial and chloroplast-aligned reads in plant ATAC-seq (Supplementary Table S1). Additionally, “quaqc” is highly efficient, requiring minimal processing power, and can process large numbers of samples simultaneously without excessive memory requirements.

## 2 Implementation

**Figure 1.**
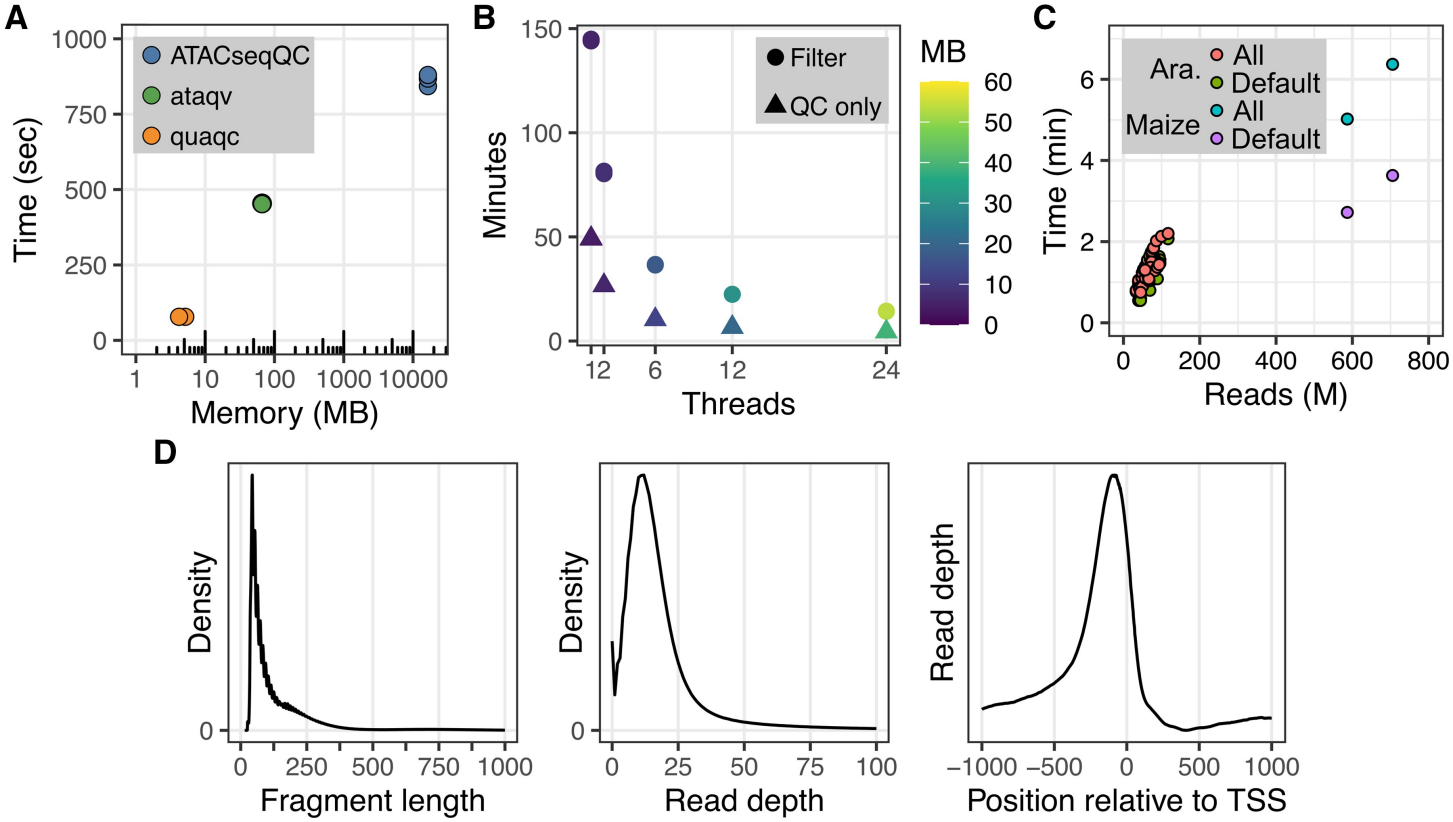
quaqc benchmarks (executed on a Linux server with dual Intel(R) Xeon(R) Gold 6130 CPU, 1535 GB RAM, and running CentOS 7.7.1908) and example outputs. (**A**) Runtime and memory usage comparison recorded using GNU Time between quaqc, ataqv, and ATACseqQC when run using a sample ATAC-seq dataset (SRR26098074). Each program was run three times. (**B**) Runtime and memory usage recorded using GNU Time when running quaqc with multithreading on a set of 50 ATAC-seq datasets (PRJNA1018553). quaqc was run either in QC only mode, or with the optional flag creating a new filtered BAM turned on. The program was run three times for each combination of parameters. (**C**) Comparison of program runtime and reads processed when using quaqc to analyze samples from the plant *Arabidopsis thaliana* (∼135 MB genome size; PRJNA1018553) as well as a plant with a larger genome, maize (∼2.4 GB genome size; SRR27443451, SRR27443454). The program was run with either default filters (Default) or the—use-all flag (All) to process all reads. (**D**) Example QC outputs from quaqc plotted using quaqcr and ggplot2 including fragment length distribution, read depth distribution, and TSS pileup, from running quaqc on a sample ATAC-seq dataset (SRR26098097).

“quaqc” is implemented using the C programming language and relies on HTSlib and Zlib functionality for reading and writing of BAM files containing aligned reads (Bonfield *et al.* 2021). These dependencies come bundled with “quaqc,” only requiring users to have a compatible C compiler such as “gcc” or “clang.” Partial support for CRAM files is also built in, though full support can be enabled by separately installing additional data compression libraries. “quaqc” performs all its functions in a single pass through a coordinate-sorted BAM file, optionally saving reads passing quality control filters into a new BAM file at the cost of additional processing time. In combination with the use of efficient data structures for cataloguing genome-wide read quality metrics, “quaqc” can process large BAMs quickly and with extremely low memory overhead in comparison to existing tools such as “ataqv” and “ATACseqQC” (Fig. 1A). Additionally, these properties allow “quaqc” to be easily parallelizable via a simple use of pthreads, reducing processing time and memory usage with an increasing number of requested parallel worker threads (Fig. 1B). Crucially, processing time is independent of genome size. Instead, the runtime scales with reads processed, allowing for rapid processing of samples from both small and large genomes (Fig. 1C).

When processing unfiltered BAMs, “quaqc” generates commonly sought-after metrics by default. Metrics can be calculated from reads aligned genome-wide, those aligned within target regions or sequences, or those aligned outside of a blacklist. They include counts and percentages of various reads by type (including duplicated reads, properly mapped paired end reads, secondary alignments, etc.) categorized by their nuclear, mitochondrial, or chloroplast origin. These categories can also contain any number of sequences, allowing for handling of incomplete assemblies, which lack a single defined mitochondrial or chloroplast sequence. All read count metrics are also emitted for high-quality nuclear reads, in addition to secondary statistics calculated from alignment size, depth, quality, GC content, as well as fragment size histograms. Optional features are also available. Providing a BED file containing peak locations will enable “quaqc” to calculate a fraction of reads in peaks score. A BED file containing transcription start site (TSS) coordinates will enable “quaqc” to generate an average read density pileup in a user-defined region around TSSs, as well as calculate a TSS enrichment score (TES). As is typically done for ATAC-seq data, the alignment position of reads is resized around the 5ʹ end of the read and optionally adjusted for the Tn5 transposition offset (Yan *et al.* 2020).

All results from “quaqc” are output in simple text format per sample, or optionally within a combined JSON file, containing detailed information about run parameters and additional statistics. The latter can be used to visualize the results by a companion R package, “quaqcr,” optionally in combination with a plotting library such as “ggplot2” (Fig. 1D) (Wickham 2016). Multiple JSON files created by “quaqc” can be used for visualization simultaneously, allowing for easy side-by-side comparison of QC metrics from different samples or individual uses of “quaqc” with alternate read filtering thresholds. A few additional special presets (or modes) are also available. For example, a footprinting mode, which, when activated, generates average single-base Tn5 transposase insertion frequency centered around transcription factor binding site coordinates from a user-provided BED file. A ChIP mode sets run parameters optimized for processing ChIP-seq data, including generating an average read pileup in target peaks instead of at TSSs. Additional modes provide parameter presets for common tasks such as filtering for likely nucleosome-free region (NFR) or nucleosome-bound region aligned reads.

While “quaqc” currently offers a comprehensive ATAC-seq quality control and read filtering feature set, future work will expand its capabilities to target group-specific reads within single BAMs (such as individual cells in the case of scATAC-seq) as well as improve its multithreading capabilities with job-stealing.

## 3 Application

ATAC-seq experiments with samples from different conditions of varying quality may require the use of optimized filters to improve their usefulness for the discovery of ACRs and quantification of genome-wide accessibility. To demonstrate the utility of *quaqc* in exploring QC metrics from diverse sample types, we downloaded and aligned reads using *bowtie2* (Langmead and Salzberg 2012) from a publicly available ATAC-seq dataset including mesophyll (SRR26098097), guard cell (SRR2609890), and root (SRR26098111) samples of the plant *Arabidopsis thaliana* (Seller and Schroeder 2023). “quaqcr” and “ggplot2” can then be used to visualize output QC data from “quaqc,” such as in this case observing the large variation in non-nuclear reads possible between plant cell types (Supplementary Fig. S1A). A closer inspection of the fragment size distributions of these three samples reveals the expected 10.5 bp periodicity resulting from tagmentation of DNA wrapped around nucleosomes (Schep *et al.* 2015) is absent from the root sample (Supplementary Fig. S1B). This may indicate the presence of free DNA in the reaction mixture for this sample, which likely explains the lower signal-to-noise ratio (Supplementary Fig. S1C).

Taking advantage of the ability to scan reads only from individual sequences or chromosomes, “quaqc” was then run repeatedly on the mesophyll sample to test a large parameter space of possible optimal filtering thresholds. We tested a total of 125 combinations of parameters for minimum MAPQ values between 0 and 40, minimum fragment lengths between 10 and 50, and maximum fragment lengths between 50 and 250 (Supplementary Fig. S1D–F). Restricting “quaqc” to reads aligned to chromosome 1 allowed us to complete this task within several minutes on a MacBook Pro M1. Then, making use of “quaqcr” and “ggplot2,” we compared the effects of these combinations on the TES (as a proxy for signal-to-noise in NFR regions) and the remaining number of reads. This revealed three key points: (i) increasing the MAPQ cutoff improves the TES with very little loss of reads (Supplementary Fig. S1D); (ii) setting a minimum fragment length cutoff has a negligible impact on the TES between 10 and 40, though a cutoff of 50 does boost it (Supplementary Fig. S1E); and (iii) reducing the maximum fragment length cutoff significantly improves the TES until 150, and below 100 all improvements are lost (Supplementary Fig. S1F).

Next, the sample BAMs were filtered using “quaqc” and the optimal parameters (minimum MAPQ of 40, minimum and maximum fragment length of 50 and 150, respectively). Peaks were called using “MACS3” (Zhang *et al.* 2008), compared with BAMs containing all nuclear reads, and loaded into the “IGV” browser (Supplementary Fig. S1G). This revealed reduced background signal in the optimized BAMs and improved peak calling, including higher numbers of peaks annotated as promoter and distal intergenic using “ChIPseeker” (Yu *et al.* 2015) (Supplementary Fig. S1H). These results clearly demonstrate the benefits of careful selection of filtering parameters for ATAC-seq data.

## Supplementary Material

btae649_Supplementary_Data
