# Supplementary material for: quaqc: efficient and quick ATAC-seq quality control and filtering
Source: Bioinformatics. 2024 Oct 30;40(11):btae649. doi: 10.1093/bioinformatics/btae649 (PMC11549019; doi:10.1093/bioinformatics/btae649)
Supplement: btae649_Supplementary_Data [file btae649_supplementary_data.pdf]

Supplementary File

quaqc: Efficient and quick ATAC-seq quality control and filtering

B.J.M. Tremblay and J.I. Qüesta

Centre for Research in Agricultural Genomics (CRAG), CSIC-IRTA-UAB, Campus UAB, Bellaterra (08193), Barcelona, Spain

Table S1

Figure S1

Table S1. Comparison of select functionality between *quaqc*, *ataqv*, and *ATACseqQC*.

Functionality	*quaqc*	*ataqv*	*ATACseqQC*
Proper handling of both mitochondria and chloroplast	✓		
Region target list	✓		✓
Blacklist	✓	✓	
Fraction of reads in peaks (FRIP)	✓	✓	
TSS enrichment score (TES)	✓	✓	✓
TSS pileup	✓	✓	✓
Specify read groups (RG)	✓*	✓*	
Mapping quality (MAPQ) histogram	✓	✓	
Read length histogram	✓		
Fragment length histogram	✓	✓	✓
Read depth histogram	✓		
GC content histogram	✓		

**ataqv* can output metrics for all individual RGs per run, whereas *quaqc* only allows for specifying sets of RGs to analyze together per run.

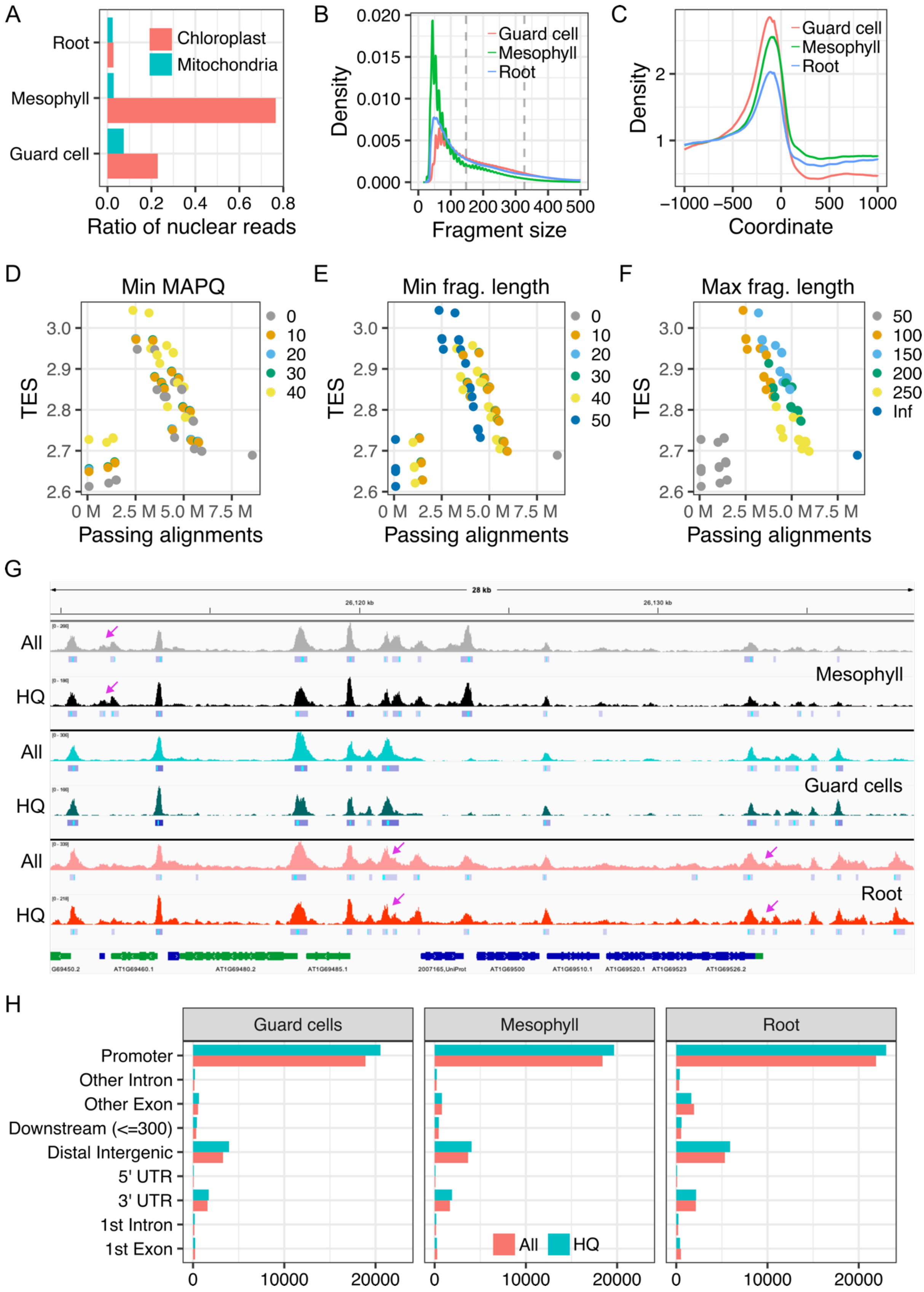

Fig. S1: Optimizing ATAC-seq signal-to-noise with *quaqc*. Three *Arabidopsis thaliana* ATAC-seq samples from mesophyll (SRR26098097), guard cell (SRR26098090), and root (SRR26098111) tissues were aligned using *bowtie2* and

analyzed using *quaqc*, making use of the *--json* flag to output a detailed report. Using the *quaqcr* package in R allows for easy exploration of various metrics such as the fraction of non-nuclear reads in the three samples, which shows the reduced chloroplast content in roots (**A**). Crucial quality control data such as fragment size distributions can also be observed, revealing possible free DNA contamination in the root sample (**B**). Likely as a result of this, the root sample has the lowest read TSS enrichment relative to the background (**C**). To test the effects of read filtering on peak calling, *quaqc* was run using the *--target-names* flag to scan reads on chromosome 1 of the mesophyll sample with various combinations of parameters. This was done once without any filters and using the *--use-all* flag (rightmost point in all plots), then all 125 possible combinations of: minimum MAPQ values 0, 10, 20, 30, and 40; minimum fragment lengths 10, 20, 30, 40, 50; and maximum fragment lengths 50, 100, 150, 200, 250. This task required only several minutes to run using a MacBook Pro M1. The QC results were saved in JSON format, then loaded into *R* using *quaqcr*. The number of chromosome 1 alignments passing filters and the TES scores were plotted using *ggplot2*. The plot is repeated three times, with colouring either for the MAPQ thresholds (**D**), minimum fragment lengths (**E**), or maximum fragment lengths (**F**). An optimal parameter combination (minimum MAPQ 40; minimum fragment length 50; maximum fragment length 150) was chosen and used to run *quaqc* to produce a new filtered BAM for each sample. *quaqc* was also used to create BAMs containing all nuclear reads run with the *--use-all* flag. These BAMs were then converted to BED format using *bedtools* and subsampled to an even depth. Peaks were subsequently called using *MACS3* with parameters *-g 116813150 -q 0.1 --nomodel --shift -37 --extsize 75 --keep-dup all.* Afterwards, all peak files and BAMs were loaded into IGV, which is shown as a screenshot of region 26,109,626-26,138,576 on chromosome 1 (**G**). This comparison reveals new peaks being detected in the high quality (HQ) BAMs created using optimized filters (purple arrows). Further comparing the number and distributions of genomic annotations of the peaks using *ChIPseeker* reveals the increased numbers of promoter and distal intergenic peaks found in the HQ BAMs as compared to BAMs containing all nuclear reads (**H**).
